# Next-Generation Analogues of AC265347 as Positive Allosteric Modulators of the Calcium-Sensing Receptor: Pharmacological Investigation of Structural Modifications at the Stereogenic Centre

**DOI:** 10.3390/ijms26062580

**Published:** 2025-03-13

**Authors:** Le Vi Dinh, Jesse Dangerfield, Aaron DeBono, Andrew N. Keller, Tracy M. Josephs, Karen J. Gregory, Katie Leach, Ben Capuano

**Affiliations:** 1 Medicinal Chemistry, Monash Institute of Pharmaceutical Sciences, Monash University, 381 Royal Parade, Parkville, VIC 3052, Australia; le.dinh@monash.edu (L.V.D.); jesse.dangerfield@monash.edu (J.D.); aaron.debono@monash.edu (A.D.); 2 Drug Discovery Biology, Monash Institute of Pharmaceutical Sciences, Monash University, 381 Royal Parade, Parkville, VIC 3052, Australia; andrew.keller@monash.edu (A.N.K.); tracy.josephs@monash.edu (T.M.J.); karen.gregory@monash.edu (K.J.G.); 3 Australian Research Council Centre for Cryo-Electron Microscopy of Membrane Proteins, 381 Royal Parade, Parkville, VIC 3052, Australia

**Keywords:** positive allosteric modulator, PAM, calcium-sensing receptor, AC265347, stereogenic centre

## Abstract

The calcium-sensing receptor (CaSR) is a validated therapeutic target in the treatment of hyperparathyroidism and related diseases. The CaSR *ago*-positive allosteric modulator (PAM), AC265347 (**1**), exhibits a chemically and pharmacologically unique profile compared to current approved CaSR PAM therapeutics. Herein, we report a series of ‘next-generation’ analogues of AC265347, investigating the impact of structural modifications at the stereogenic centre on CaSR PAM activity. Compounds **5** and **7b** featuring the alcohol functional group showed *ago*-PAM profiles comparable to **1**, whilst compounds **6**, **7** and **9** devoid of this functionality were ‘pure’ PAMs with no intrinsic agonism. These novel chemical tools provide an opportunity to explore the therapeutic potential of AC265347-like PAMs as a function of affinity, cooperativity and intrinsic agonism.

## 1. Introduction

**Figure 1:**
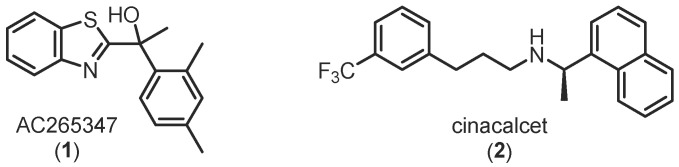
Chemical structures of two chemical classes of CaSR positive allosteric modulators (PAMs): benzothiazole (AC265347, **1**) and naphthylalkylamine (cinacalcet, **2**) [3].

AC265347 (**1**) (Figure 1), identified by ACADIA Pharmaceuticals from a screening library, is composed of a benzothiazole scaffold, a stereogenic centre bearing a tertiary alcohol and a dimethyl-substituted phenyl motif. This molecule is a positive allosteric modulator of the calcium-sensing receptor (CaSR) with intrinsic agonism (*ago*-PAM), and structurally distinct from the typical naphthylalkylamine class of CaSR modulators (e.g., cinacalcet (**2**), Figure 1). The initial in vivo study used the racemic mixture of AC265347 and demonstrated potent suppression of PTH release in rodents, consistent with a CaSR PAM profile [1]. Notably, the *S*-enantiomer had 10-fold greater potency than the *R*-enantiomer [1,2]. Further analysis confirmed AC265347’s binding selectivity for the CaSR, with no off-target activation at the GABAb receptors and PTH1 receptors, thus validating its therapeutic potential [1]. The evaluation of AC265347 as a CaSR therapeutic was discontinued for undisclosed reasons, with the last publication in 2011. However, this unique molecule warrants further investigation based on its *ago*-PAM activity accompanied by an improved side-effect profile.

The work described herein is an extension of our previously published research [4] and primarily focused on implementing structural modifications at the stereogenic centre of AC265347 (**1**) and elucidating the ensuing pharmacology. We sought to provide further understanding of the key attributes of the active motif to guide new rational drug design towards generating lead drug candidates at this important therapeutic target for treating CaSR-related disorders. Our efforts used the recently published cryo-EM CaSR structures to inform and rationalise medicinal chemistry efforts towards the development of compounds with improved CaSR activity, specifically: (i) affinity of the allosteric ligand for the free receptor (*K*_B_); cooperativity factors defining the magnitude and direction of the allosteric ligand’s effect on the orthosteric ligand’s affinity (*α*) and/or downstream efficacy (*β*); and the intrinsic agonist efficacy of the allosteric ligand (*τ*_B_) [5,6,7].

**Figure 2:**
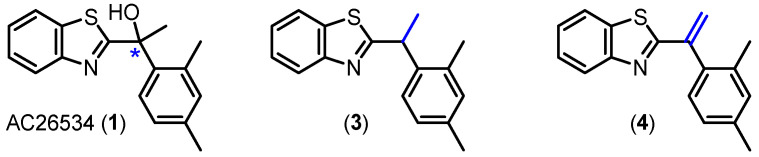
Previous structural modifications at the stereogenic centre (*) of AC265347 (**1**), namely the de-oxy analogue (**3**) and the exo-methylene analogue (**4**), investigating the importance of H-bonding interactions.

The work of Gustaffson et al. [2] examined the importance of the hydroxy group at the stereogenic centre by completely removing the H-bonding functional group, as depicted in analogues **3** and **4** (Figure 2). Upon investigation, the sp^3^-hybridised analogue **3** was reported to be approximately 100-fold less potent than AC265347, while the sp^2^-hybridised olefinic analogue **4** lost all activity at the CaSR. Conclusively, the hydroxy group was deemed crucial for activity, and suspected to be involved in weak H-bond interactions [2]. However, previous AC265347 hydroxy group exploration was limited to complete removal of the H-bonding functional group [2]. To extend the previous findings, we sought to diversify the current structure-activity relationship (SAR) library by making structural modifications targeting the hydroxy group.

**Figure 3:**
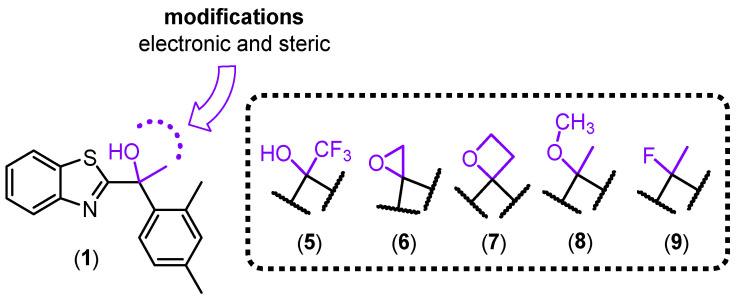
Proposed structural modifications to the hydroxy group at the stereogenic centre of AC265347 (**1**) revealing the trifluoromethyl (**5**), epoxide (**6**), oxetane (**7**), methoxy (**8**) and fluoro (**9**) analogues.

Herein, we aimed to delineate the role of H-bond acceptor and donor predicted to partake in binding to the CaSR. The suite of analogues (Figure 3) includes subtle structural modifications to the OH group, for example, by substitution with a methoxy group (**8**). This modification removes the H-bond donor property, thus allowing examination of the H-bond acceptor ability alone. Bioisosteric replacement of the OH group with fluorine (**9**) is commonly used in medicinal chemistry because of its similarity in size, polarity and electronegativity between oxygen and fluorine atoms [8,9,10]. The substitution strategy is thought to provide metabolic stability, mainly due to the high C–F bond energy and conformational disturbance to avoid recognition by enzymes [11,12,13,14]. The replacement of OH for F is generally restricted to the H-bond acceptor ability, which thus allows examination of the H-bond donor property provided by the hydroxy hydrogen atom. Additionally, the epoxide (**6**) [15] and oxetane (**7**) [16] were synthesised to challenge the importance of spatial orientation and rotational freedom on PAM activity at the CaSR. While the oxetane derivative angles the bonds almost perpendicularly at ~90°, the epoxide derivative is more constrained, with a bond angle at 60° (Figure 3), thereby bringing the O and C atoms in significantly closer proximity compared to the parent compound (~105°) and increasing the relative distance between the aromatic motifs. We also included a previously published analogue (**5**) [4] whereby the methyl substituent at the stereocentre was replaced with trifluoromethyl to enable further comparisons. We anticipate changes in allosteric effect with these structural changes, thus revealing the importance in maintaining the rotational freedom at the stereogenic centre and allowing us to dissect the role of H-bond donor/acceptor properties.

By generating a focused library of analogues with rational structural modifications on lead compound **1**, we sought to delineate the structural motif responsible for affinity, cooperativity or agonism at the CaSR. Analogues were subjected to Ca^2+^_i_ mobilisation assays and the activity quantified using the operational model of agonism and allosteric modulation (refer to Section 3.3). Affinity, cooperativity and agonism were extracted and compared to the reference compounds **1** and **5**. Herein, we describe the synthetic strategies, characterisation and pharmacological evaluations of the designed analogues.

## 2. Results and Discussion

### 2.1. Chemistry

Our SAR study focused on investigating the importance of the H-bond donor and acceptor properties of parent compound **1**. To further address the limitation in our understanding, we removed the H-bond donor component by replacing the hydroxy group with methoxy, fluorine, epoxide and oxetane as H-bond acceptor moieties. The employment of epoxide (**6**) and oxetane (**7**) motifs would challenge the spatial importance of the H-bond acceptor and the methyl group at the stereogenic centre with their intrinsic rigidity. The methoxy substitution (**8**) simply provided greater flexibility compared to **6** and **7**, however structurally bulkier than a hydroxy group. Replacing the hydroxy group with a fluorine (**9**) though this retained H-bond acceptor ability, satisfied steric bulk and electronegativity, and the differences between oxygen and fluorine atoms could result in a different pharmacological profile. The literature compound, (**5**), was included into this study as it evaluated the biochemical effect of isosteric replacement of the methyl substituent for trifluoromethyl at the stereogenic centre. Herein, we describe the synthetic pathways and challenges encountered in the generation of analogues that examine the importance of the hydroxy group in **1**.

**Scheme 1:**
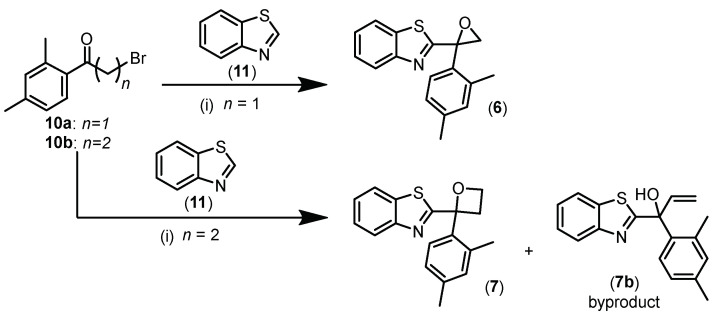
*^a^* Synthesis of *O*-cyclised analogues epoxide **6** and oxetane **7**. *^a^* Reagents and conditions: (i) *n*BuLi, THF, N_2_, −78 °C (**6** (40%), **7** (9%) and **7b** (6%)).

The *O*-cyclised series synthesis contained two analogues, epoxide **6** and oxetane **7** (Scheme 1), to examine the effect of removing the H-bond donor property and the importance of spatial orientation by introducing rigidity to the compound structure. The reaction proceeded with the lithiated benzothiazole species generated in situ as a nucleophile attacking the carbonyl carbon of selected electrophiles [17]. The intramolecular cyclisation resulted in epoxide **6** and oxetane **7** analogues.

Characteristic proton splitting of epoxide hydrogens of **6** at δ 3.4 and 3.7 ppm as doublets, each integrating to one proton, was observed as anticipated. The small coupling constant of 5.9 Hz for geminal protons supported the formation of the epoxide product **6**. HRMS analysis found *m*/*z* 282.0959, which reinforces the molecular formula C_17_H_15_NOS and identity of **6**.

**Scheme 2:**
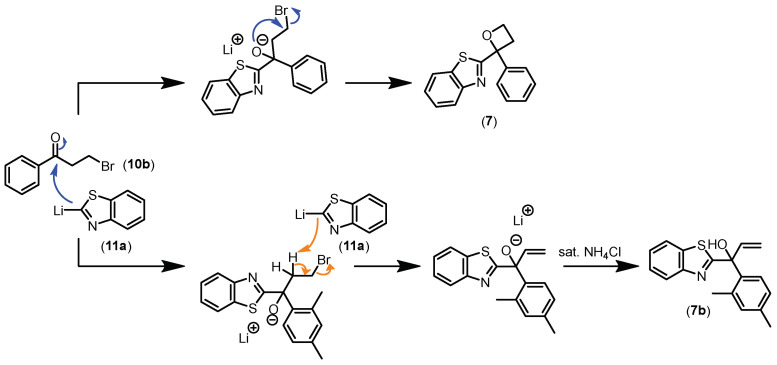
Proposed mechanism for the cyclisation of oxetane **7** and allylic alcohol by-product **7b**.

While nucleophilic addition occurred between the lithiated benzothiazole (**11a**) and the selected carbonyl carbon followed by *O*-cyclisation with bromide ion as the leaving group to form oxetane **7** (as illustrated in Scheme 2), a significant by-product corresponding to the terminal alkene **7b** was also detected. Besides acting as an intended nucleophile, **11a** can also act as a base, thereby deprotonating the α-hydrogen. This would subsequently allow the formation of a terminal alkene via elimination, resulting in the allylic alcohol **7b** product, thereby preventing the desired intramolecular cyclisation.

Our initial column purification attempt collected both oxetane **7** and allylic alcohol **7b** due to their small difference in *R_f_* values. This prompted a change in purification strategy, to which preparative thin-layer chromatography (TLC) was applied, followed by characterisation of oxetane **7** and allylic alcohol **7b**. Distinctive signals corresponding to oxetane methylene hydrogen atoms H3 and H4 in the ^1^H NMR spectrum were observed, whereby all protons on the oxetane ring are non-equivalent because of the stereogenic centre. In addition, DEPT ^13^C NMR analysis found C3 and C4 at 34 and 67 ppm, respectively, as secondary carbon signals, thus reinforcing the successful formation of oxetane **7**. Characterisation of allylic alcohol **7b** found terminal alkene ^1^H NMR resonances at δ 6.6, 5.5 and 5.4 ppm as doublet-of-doublets integrating to one proton each. The highly deshielded signals observed in the spectrum are characteristic of alkene protons, convincing evidence to support the formation of by-product **7b** [18]. The generation of epoxide **6** and oxetane **7** concluded the series of *O*-cyclised analogues, allowing us to examine the effect of installing rigid groups at the stereogenic centre and the importance of the H-bond donor property. Additionally, we address the importance of H-bond interactions by simple substitution of the hydroxy (**1**) with a methoxy group (**8**) and fluorine substituent (**9**). While the simple modification of methoxy **8** removed the H-bond donor effect, fluorine **9** maintains H-bond acceptor ability, but carries a different electronegativity compared to the oxygen atom.

**Scheme 3:**
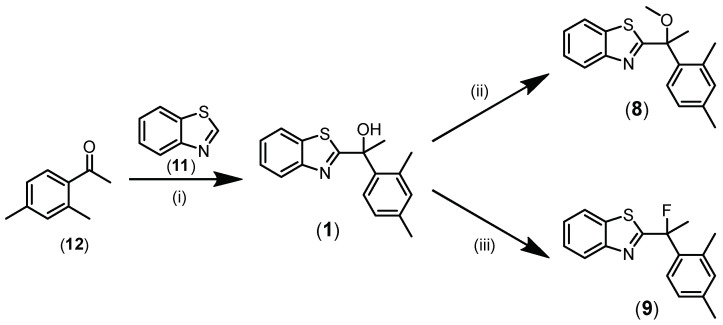
*^a^* Synthesis of the methoxy (**8**) and fluoro (**9**) analogues directly from AC265347 (**1**), to investigate the biochemical effect of compounds without H-bond donor properties. *^a^* Reagents and conditions: (i) *n*BuLi, THF, N_2_(g), −78 °C, 70%; (ii) NaH, MeI, THF, 50%; (iii) XtalFluor-M, DBU, dry DCM, −78 °C to rt, 12%.

The synthesis of *O*-alkylated **8** began with AC265347 generated in-house utilising commercially available acetophenone and benzothiazole. The *O*-alkylation reaction employed sodium hydride (NaH) as a base to deprotonate the hydroxy group, to allow for nucleophilic attack of the methyl iodide (MeI) to take place (Scheme 3) [19]. This reaction resulted in a low conversion of 25–30%, with the addition of 1 equivalent of NaH and 2 equivalents of MeI. Thus, 2 and 2.5 equivalents of NaH and MeI, respectively, were used to optimise the conversion of product to 50% by HPLC. This transformation was supported by ^1^H NMR in the absence of the hydroxy proton signal previously observed at δ 3.7 ppm (1H) for alcohol **1** and the presence of a downfield singlet resonance at δ 3.2 ppm (3H) for the methoxy group of **8**.

The synthesis of **9** utilised the safer XtalFluor-M over the commonly used but hazardous DAST and Deoxo-Fluor as deoxofluorinating agent (Scheme 3). DBU was employed as a preferred promoter to minimise formation of dimerised ether and sulfinate by-products [20,21]. The resulting crude reaction product suggested a minor conversion of product evident on LCMS over the course of 18 h. A gradient column of straight toluene followed by 20:1 toluene:EtOAc furnished the desired product **9** in a low yield of 12%. To confirm the identity of the newly formed product, we performed ^19^F NMR alongside ^1^H NMR, ^13^C NMR and HRMS experiments. The ^19^F NMR spectrum reaffirmed the presence of fluorine atom within the compound structure at δ −96 ppm as a singlet. Additionally, the absence of the characteristic hydroxy signal at δ 3.7 ppm on the ^1^H NMR spectrum pointed to successful deoxofluorination of alcohol **1** to produce fluorinated compound **9**. A large coupling constant is typically reported for the C–F bond in the ^13^C NMR spectrum, which is consistent with our observation of alkyl fluoride **9** at δ 98 ppm as a doublet (*J* = 172 Hz). Carbon atoms two bonds away from fluorine expectedly displayed smaller coupling constants (21–30 Hz). Smaller coupling constants for carbon atoms three or more bonds away were also observed on the ^13^C NMR spectrum of alkyl fluoride **9** (see experimental section for more detail).

### 2.2. Pharmacology

**Figure 4:**
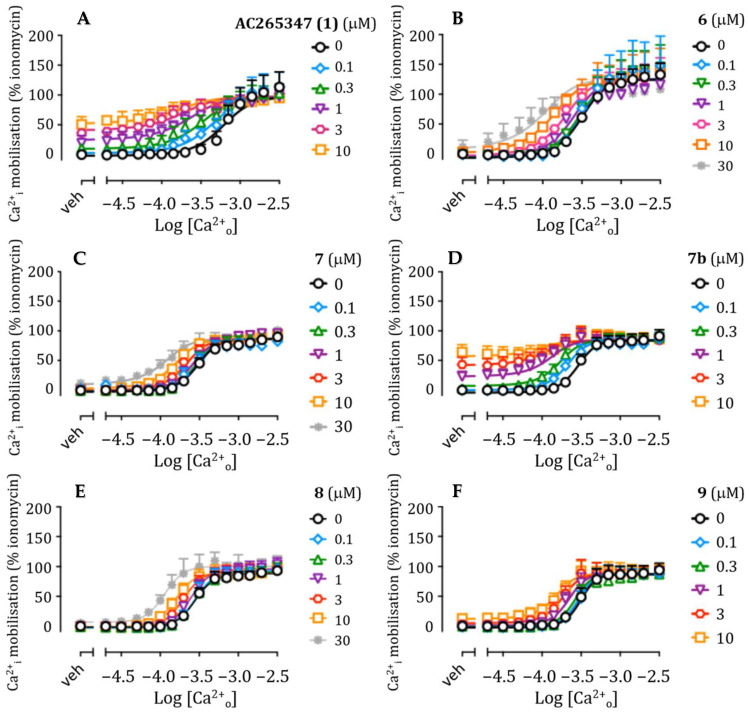
Pharmacological evaluation of AC265347 (**1**, panel (**A**)) and analogues for PAM activity using Ca^2+^_i_ mobilisation assays. Concentration–response curves are shown for Ca^2+^_o_ in the absence or presence of various concentrations of epoxide **6** (panel (**B**)), oxetane **7** (panel (**C**)), allylic alcohol by-product **7b** (panel (**D**)), methoxy **8** (panel (**E**)) and fluoro **9** (panel (**F**)) compared to parent compound AC265347 (**1**) (panel (**A**)). Data are mean ± SD pooled from n ≥ 3 independent experiments performed in duplicate. Curves through the data points are the best fit of Equation (1) (refer to Section 3.3.2) to the data. NB: None of the estimates were significantly different from that derived for AC265347 (**1**), *p* < 0.05 in one-way ANOVA Dunnett’s multiple-comparison post hoc test.

Each of the synthesised compounds was tested as the racemic mixture and assessed for modulation of Ca^2+^_o_-stimulated Ca^2+^_i_ mobilisation in recombinant HEK293 cells stably expressing inducible wild-type human CaSR (FlpIn-Trex-HEK293-CaSR) (Figure 4) to determine their affinity (*K*_B_) for the allosteric site, cooperativity effect on the orthosteric ligand’s affinity (*α*) and/or efficacy (*β*) and potential intrinsic agonism (*τ*_B_) (Table 1) [5] (a summary of the parameters from fitting the operational model of comparative co-operative agonism and allosterism to the collated data from multiple independent ex-periments can be found in Supplementary Table S1 in the Supporting Information). Figure 4A depicts the concentration–response curve for the reference racemic compound AC265347 (**1**), demonstrating (i) the characteristic leftward shift with increasing concentrations of **1**, which is indicative of positive allosteric modulation (cooperativity) of the endogenous orthosteric ligand Ca^2+^, and (ii) the characteristic upward shift with increasing concentrations of **1** in the absence of Ca^2+^ (vehicle), indicative of allosteric agonism; hence the classification of **1** as an *ago*-PAM.

Upon removal of the H-bond donor ability and constraining the rotational freedom of oxygen as an epoxide to generate compound **6**, a modest reduction in affinity, comparable cooperativity and loss of intrinsic agonism was observed compared to **1**. The prominent reduction in agonism was evident by the lack of baseline increase in the Ca^2+^_o_ concentration–response curve (Figure 4B). A similar trend was observed for oxetane-containing derivative **7**. The data collected from both epoxide **6** and oxetane **7** (Table 1, Figure 4B,C) suggest low tolerability of a more constrained spatial orientation. Disruption of the potential intramolecular interaction between the alcohol O and neighbouring benzothiazole S atoms may also contribute to the reduced activity. Interestingly, novel compound **7b**, discovered as a by-product during the synthesis of **7**, retained affinity, and exhibited a trend towards increased cooperativity and agonism compared to **1** (Figure 4D). Substituting the methyl with an ethenyl group in **7b** did not hinder hydroxy group rotational freedom, which potentially retained CaSR allosteric activity. This feature was also observed with our previously published derivative (**5**), containing the CF_3_ as a substitute for CH_3_ whilst maintaining the OH group, and exhibiting an *ago*-PAM profile (Table 1) comparable to **1**.

The structural modification resulting from replacing the hydroxy group with the methoxy group, in **8**, could not be derived (Table 1, Figure 4E). The interaction between compound **8** and Ca^2+^_o_ could not reliably be fitted with the operational model of cooperative agonism and allosterism. Therefore, these data are presented with a four-parameter sigmoidal concentration–response curve fitted to each data set, where the Hill slope is shared for all data sets (3.5 ± 0.3). Notably, in the presence of compound **8** at 10 μM, the pEC_50_ of Ca^2+^_o_ increased 1.8-fold relative to the vehicle (from 3.55 ± 0.04 to 3.81 ± 0.03), with no apparent intrinsic agonist activity. In comparison, in the presence of AC265347 (**1**) at 10 μM, the pEC_50_ of Ca^2+^_o_ increased 7.6-fold (from 3.06 ± 0.05 to 3.94 ± 0.13). The apparent trend in cooperativity devoid of agonism may be a consequence of removing the H-bond donor properties.

Replacement of the hydroxy group with the fluoro substituent was supported by the ability of fluorine to make H-bond interactions that mimic those of the hydroxy group. Fluorine is known as a bioisostere of OH with similarity in polarity and size [22]. Furthermore, fluorine is hydrophobic compared to oxygen, and therefore its inclusion can enhance oral bioavailability and display a more druglike profile [23,24]. Given the similarities shared between a hydroxy group and fluorine, fluorinated compound **9** saw a comparable affinity and commensurate level of cooperativity compared to **1** (Table 1, Figure 4F), and was virtually devoid of intrinsic agonism. AC265347 and the derivatives reported herein (except for **8**) have degrees of cooperativity (log*αβ*) ranging from 0.9 to 2.2, comparable to reported cooperativity factors for clinically used CaSR PAMs cinacalcet and evocalcet, log*αβ* factors of 1.4 and 1.8, respectively [25]. The new derivatives described herein largely have reduced intrinsic agonist activity, exhibiting ‘pure’ PAM activity. Such investigations and exploration of SAR within a discovery program provide a means to evaluate the relative contribution of cooperativity versus intrinsic efficacy to engendering a desired therapeutic effect. In addition to CaSR PAMs, the clinical calcimimetic, etelcalcetide, is a direct CaSR allosteric agonist [26]. Whether or not intrinsic CaSR agonist activity or pure PAM activity is most desirable for any given indication remains an open question. We must continue to build our understanding of the drivers of therapeutic efficacy when targeting the CaSR for different conditions or in each individual person. Overall, compounds **6**, **7** and **9**, although displaying a range of affinities (10–123 μM) and cooperativities (log*αβ* ranging from 1.45–1.95), did exhibit a relatively ‘pure’ PAM profile devoid of intrinsic agonism.

**Table 1:** SAR surrounding the AC265347 hydroxy group and pharmacological profiles of compounds. Functional affinity (p*K*_B_), positive allosteric cooperativity (*αβ*) and intrinsic (allosteric) agonism (*τ*_B_) were determined using Ca^2+^_i_ mobilisation assays in FlpIn-Trex-HEK293-CaSR cells. Data are mean ± SEM estimated from at least three independent experiments (n) performed in duplicate.

Entry	Structure	p*K*_B_ ± SEM (*K*_B,_ μM)	Log*αβ* ± SEM (*αβ*)	Log*τ*_B_ ± SEM (*τ_B_*)	n
AC265347 (**1**) *^a^*	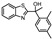	5.47 ± 0.11 (3.4)	1.63 ± 0.16 (43)	0.12 ± 0.08 (1.3)	5
**5** *^a^*	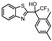	5.30 ± 0.23 (5.0)	0.94 ± 0.27 (8.7)	0.53 ± 0.18 (3.4)	5
**6**	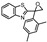	4.54 ± 0.49 (29)	1.45± 0.41 (28)	ND *^b^*	3–7
**7**	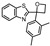	3.91 ± 0.66 (123)	1.95 ± 0.61 (89)	−0.29 ± 0.54 (0.5)	3–7
**7b**	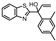	5.09 ± 0.27 (8.1)	2.24 ± 0.37 (174)	0.58 ± 0.20 (3.8)	4
**8** *^c^*	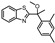	ND *^b^*	ND *^b^*	ND *^b^*	3
**9**	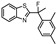	4.98 ± 0.41 (10)	1.59 ± 0.37 (39)	−0.43 ± 0.26 (0.4)	4

*^a^* Reanalysis of previously published data sets [4] using [27] Equation (1) herein; see Section 3.3.2. For these analyses, all parameters were allowed to float and derived from global fit of collated experiments; *^b^* ND denotes ‘not determined’ due to either no apparent agonism or an ambiguous fit of the collated data set. NB: None of the estimates were significantly different from that derived for AC265347 (**1**), *p* < 0.05 in one-way ANOVA Dunnett’s multiple-comparison post hoc test; *^c^* the interaction between **8** and Ca^2+^_o_ could not reliably be fitted with the operational model of cooperative agonism and allosterism. Data are presented with a four-parameter sigmoidal concentration–response curve fitted to each data set, where the Hill slope is shared for all data sets (3.5 ± 0.3).

### 2.3. Molecular Docking Study

**Figure 5:**
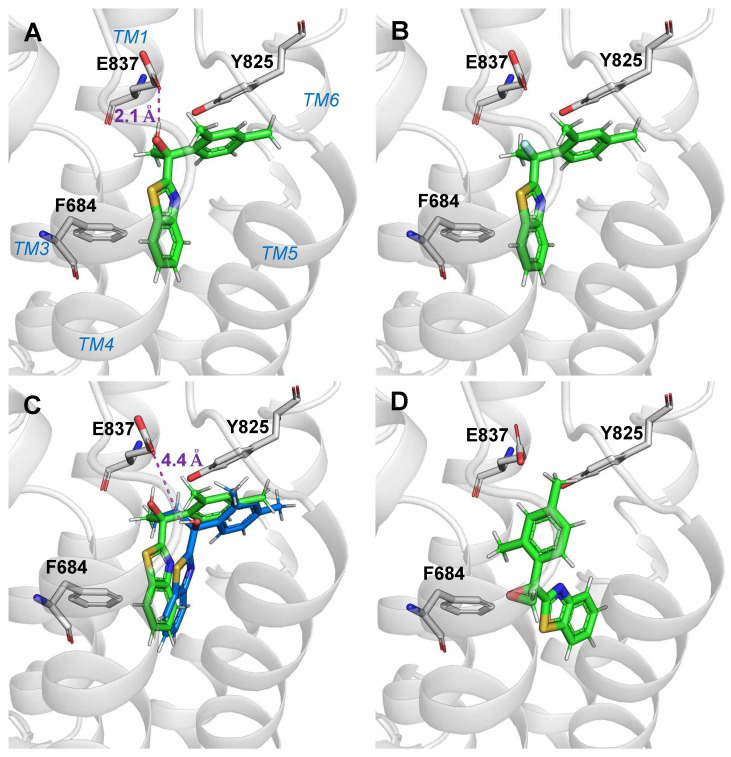
Proposed binding mode of representative compounds: (**A**) AC265347 (**1**), (**B**) compound **9**, (**C**) overlay of AC265347 (**1**) and compound **7b** with a distance of 1.9 Å between the stereogenic carbon centres and (**D**) compound **6** in the CaSR 7TM in an active model derived from the Cryo-EM structure PDBID:7M3G [30]. The receptor is shown in cartoon form, depicting the α-helices in light grey. Key amino acid residues (F684, TM3; Y825, TM6 and E837, TM7) and the corresponding ligand are shown in sticks form, with the ligand hydrogens shown to highlight predicted hydrogen bond interactions. The chemical structure representation is as follows: carbon (grey for protein and green for ligands); nitrogen (blue); oxygen (red); hydrogen (white); sulphur (yellow); fluorine (turquoise).

Currently, all known small molecule modulators of the CaSR bind to an allosteric pocket within the seven-transmembrane domain. The amino acid E837 is of particular interest, as it is a critical residue for the activity of the arylalkylamine class of CaSR modulators that includes cinacalcet [28]. Previous work by Gustaffson et al. [2], in addition to in-house modelling, suggested AC265347 forms a weak hydrogen bond interaction with E837. Molecular docking was undertaken to identify whether the structural changes in the prepared AC265347 analogues could be linked to a difference in binding conformation that would relate to the pharmacological characteristics observed. Currently, there is no structure solved with AC265347 bound to CaSR, so predictive modelling is limited to using a model derived from a Cryo-EM structure with the PAM cinacalcet bound (PDBID:7M3G) in conjunction with mutagenesis data previously published [29,30]. Our modelling predicts that AC265347 (Figure 5A) binds towards the top of the seven-transmembrane domain, with the benzothiazole core oriented towards the bottom of the domain and the dimethyl-substituted phenyl motif extending towards extracellular space. The docking indicates the alcohol functional group of the more active *S*-enantiomer (eutomer) of AC265347 is oriented towards and predicted to interact with E837 via a hydrogen bond, while the alcohol of the less active *R*-enantiomer (distomer) faces away from E837. The difference in conformation and associated molecular interactions may account for the 10-fold increased activity of the AC265347 eutomer. The binding poses of the trifluoromethyl (**5**), allylic alcohol (**7b**, Figure 5C) and methoxy analogues (**8**) match the orientation of the AC265347 eutomer; however, their positions are slightly displaced due to increased steric hinderance and/or electronic effects at the stereocentre (e.g., there is a distance of 1.9 Å between the stereogenic carbon centres of compounds **1** and **7b**; Figure 5C). The epoxide (**6**, Figure 5D) and oxetane (**7**) variants are both positioned so that the stereocentre is towards the left side of the binding pocket near L776 and F684 and therefore facing away from E837. The binding pose of compound **9**, with the tertiary alcohol substituted for a fluorine (Figure 5B), matches that of AC265347 due to their similar molecular sizes. Our findings show compounds (**6**, **7**, **8** and **9**) that are incapable of forming the predicted hydrogen bond with E837 result in reduced or complete loss of intrinsic agonism. This is consistent with the previously published mutagenesis data highlighting the importance of this residue [29]. This docking study suggests the hydrogen bond capability of the tertiary alcohol at the stereocentre is important for the intrinsic agonism of this chemotype.

## 3. Materials and Methods

### 3.1. Materials

#### 3.1.1. General Chemistry

All solvents and chemicals were purchased from standard suppliers and were used without any further purification (Merck Life Science Pty Ltd., Melbourne, Victoria, Australia; Combi-Blocks, San Diego, CA, USA; Ambeed Inc., Arlington Heights, IL, USA; Aaron Chemicals, San Diego, CA, USA; Matrix Fine Chemicals, Flums, Switzerland, EU). ^1^H NMR and ^13^C NMR spectra were acquired at 400.13 and 100.62 MHz, respectively, on a Bruker Avance III Nanobay 400 MHz NMR spectrometer (Billerica, MA, USA) coupled to the BACS 60 automatic sample changer and equipped with a 5 mm PABBO BB-1H/D Z-GRD probe. All spectra obtained were processed using MestReNova software (v.6.0) (Barcelona, Spain, EU). Chemical shifts (δ) for all ^1^H NMR spectra are reported in parts per million (ppm) using deuterated solvents as reference [31,32,33]. The data for all spectra are reported in the following format: chemical shift (δ), (multiplicity, coupling constants *J* (Hz), integral), where the multiplicity is defined as s = singlet, d = doublet, t = triplet, q = quartet, p = pentet and m = multiplet. ^13^C NMR were routinely carried out as *J*-modulated spin-echo (JMOD) or Distortionless Enhancement by Polarisation Transfer (DEPT) experiments, and all ^13^C NMR δ are reported in ppm. Thin-layer chromatography (TLC) was carried out routinely on silica gel 60F254 precoated plates (0.25 mm, Sigma-Aldrich, St. Louis, MO, USA). Flash-column chromatography was carried out using Davisil LC60A silica gel, 40–63 μm.

Liquid chromatography–mass spectrometry (LC–MS) was performed on one of two instruments, either an Agilent 6100 Series Single Quad LC/MS (Santa Clara, CA, USA) or an Agilent 1200 Series HPLC (equipped with a 1200 Series G13111A Quaternary Pump, G1329A Thermostatted Autosampler and a G1314B Variable Wavelength Detector) (Santa Clara, CA, USA), and the data were processed using LC/MSD Chemstation Rev.B.04.01 SP1 coupled with Easy Access B.04.00 software. Both systems were equipped with a Reverse Phase Luna C8(2) (5 μm, 50 × 4.6 mm, 100 Å) column maintained at 30 °C. An MeCN gradient (5–100%) was used to obtain optimal separation, where 4 min were required for the gradient to reach 100% MeCN, after which it was maintained for a further 3 min before requiring 3 min to return to the initial gradient of 5% MeCN (total run time = 10 min). Solvent A = 0.1% aqueous formic acid; Solvent B = MeCN/0.1% formic acid.

The purity and retention time of final products were determined using analytical HPLC and high-resolution mass spectrometry (HRMS). Analytical HPLC was carried out using an Agilent 1260 Infinity Analytical HPLC (Santa Clara, CA, USA) fitted with a Zorbax Eclipse Plus C18 Rapid Resolution column (100 mm × 4.60 mm, 3.5 μm) using a binary solvent system: solvent A of 0.1% aqueous TFA; solvent B of 0.1% TFA in MeCN. Gradient elution was achieved over 10 min using 95% A + 5% B, then brought to 100% B over 9 min, and 100% B was maintained for 1 min at a flow rate of 1 mL/min monitored at both 214 and 254 nm. HRMS was conducted on an Agilent 6224 TOP LC/MS Mass Spectrometer coupled to an Agilent 1290 Infinity (Santa Clara, CA, USA). All data were acquired and reference mas corrected via dual-spray electrospray ionisation (ESI) source. Each scan or data point on the total ion chromatogram (TIC) is the average of 13,700 transients, producing one spectrum per second. Mass spectra were created by averaging the scans across each peak and background subtracted against the first 10 s of the TIC. Data acquisition was carried out using the Agilent Mass Hunter Data Acquisition software version B.05.00 Build 5.0.5042 (Santa Clara, CA, USA), and analysis was performed using Mass Hunter Qualitative Analysis version B.05.00 Build 5.0.519.13 (Santa Clara, CA, USA).

#### 3.1.2. Cell Culture

Flp-In^TM^ TREX^TM^ Human Embryonic Kidney (HEK) 293 cells were purchased from Invitrogen (Carlsbad, CA, USA). Fluo-8-AM (acetoxymethyl ester) was purchased from Abcam (Cambridge, MA, USA). Dulbecco’s Modified Eagle’s Medium (high glucose), poly-D-lysine, hygromycin B, blasticidin HCl, tetracycline and all other reagents were purchased from Sigma Aldrich (St. Louis, MO, USA). Ionomycin was purchased from Cayman Chemicals (Ann Arbor, MI, USA).

FlpIn HEK293 TRex cells stably expressing cmyc-tagged WT CaSR were grown in DMEM supplemented with 5% FBS and antibiotic selection (200 μg/mL hygromycin; 5 μg/mL blasticidin). All cells were maintained at 37 °C in a 5% CO_2_ humidified incubator, grown to confluence in the presence of 100 ng/mL tetracycline to induce CaSR expression, and then seeded in 96-well culture plates.

### 3.2. Chemical Synthesis of Compounds

*General procedure A [34] (lithiation)*

To a solution of benzothiazole (**11**, 1.1 equiv.) in dry THF (3 mL) at −78 °C was added *n*BuLi solution (2.5 M, 1.3 equiv.) in a dropwise fashion and stirred for 1 h. A precooled substituted acetophenone (1 equiv.) in dry THF solution was subsequently added dropwise to the reaction mixture, then allowed to reach r.t. and stirred for a further 24 h. The resulting mixture was quenched by saturated NH_4_Cl (5 mL) and extracted with EtOAc (3 × 10 mL) and then washed with saturated brine. The combined organic layer was dried over anhydrous Na_2_SO_4_, then concentrated under reduced pressure.

*General procedure B [17] (O-cyclisation)*

Preparation of lithiobenzothiazole (**11a**) was carried out in accordance with general procedure A. To a solution of lithiobenzothiazole was added halo-substituted ketone (0.7 equiv. to benzothiazole) in dry THF (5 mL) at −78 °C. The reaction mixture was allowed to warm to r.t. over an hour and stirred for another 5 h. The resulting mixture was quenched with sat. NH_4_Cl (5 mL), extracted with EtOAc, washed with brine, dried over Na_2_SO_4_ and evaporated under vacuum.

*Synthesis of 1-(Benzo[d]thiazol-2-yl)-1-(2,4-dimethylphenyl)ethan-1-ol (* **1** *) [4]*

Synthesis occurred as described in general procedure A. Purification using silica gel column chromatography (5% acetone in toluene) was performed to afford the product as white crystal (133 mg, 70%). ^1^H NMR (401 MHz, CDCl_3_) δ 8.01 (d, *J* = 8.2 Hz, 1H), 7.81 (d, *J* = 8.0 Hz, 1H), 7.54 (d, *J* = 8.0 Hz, 1H), 7.51–7.45 (m, 1H), 7.37 (t, *J* = 7.6 Hz, 1H), 7.07 (d, *J* = 7.9 Hz, 1H), 6.98 (s, 1H), 3.73 (s, 1H), 2.33 (s, 3H), 2.14 (s, 3H), 2.11 (s, 3H). ^13^C NMR (101 MHz, CDCl_3_) δ 179.95 (C), 152.26 (C), 138.76 (C), 138.53 (C), 137.66 (C), 135.93 (C), 133.47 (CH), 126.45 (CH), 126.32 (CH), 126.25 (CH), 125.30 (CH), 123.16 (CH), 121.88 (CH), 31.33 (CH_3_), 21.35 (CH_3_), 21.06 (CH_3_). HRMS (*m*/*z*): C_17_H_17_NOS requires [M+H]^+^ 284.1104; found 284.1115.

*Synthesis of 2-(2-(2,4-Dimethylphenyl)oxiran-2-yl)benzo[d]thiazole (* **6** *)*

Synthesis occurred as described in general procedure B. The resulting crude was purified via silica gel column chromatography (8:1 petroleum ether:EtOAc) to afford the product as a light-yellow paste (89 mg, 40%). ^1^H NMR (401 MHz, CDCl_3_) δ 8.00 (ddd, *J* = 8.2, 1.2, 0.7 Hz, 1H), 7.83 (ddd, *J* = 7.9, 1.3, 0.6 Hz, 1H), 7.49–7.42 (m, 2H), 7.37 (ddd, *J* = 8.3, 7.2, 1.2 Hz, 1H), 7.14–7.10 (m, 1H), 7.09–7.05 (m, 1H), 3.71 (d, *J* = 5.9 Hz, 1H), 3.44 (d, *J* = 5.9 Hz, 1H), 2.38 (s, 3H), 2.35 (s, 3H). ^13^C NMR (101 MHz, CDCl_3_) δ 171.89 (C), 153.77 (C), 139.22 (C), 137.86 (C), 135.50 (C), 132.17 (C), 131.43 (CH), 128.76 (CH), 126.65 (CH), 126.17 (CH), 125.34 (CH), 123.58 (CH), 121.69 (CH), 60.25 (C), 57.60 (CH_2_), 21.38 (CH_3_), 19.59 (CH_3_). HPLC: *t*_R_ 6.027 min, >95% purity (214 and 254 nm). HRMS (*m*/*z*): C_17_H_15_NOS requires [M+H]^+^ 282.0947; found 282.0959.

*Synthesis of 2-(2-(2,4-Dimethylphenyl)oxetan-2-yl)benzo[d]thiazole (* **7** *)*

Synthesis occurred as described in general procedure B. Purification using silica gel column chromatography (10:1 petroleum ether:EtOAc) afforded product mixture **7** and **7b**. Subsequently, the preparative TLC purification method (40:1 toluene:EtOAc) separated **37** (14 mg, 9%) as a clear oil and **7b** as a white solid (9 mg, 6%). ^1^H NMR (401 MHz, CDCl_3_) δ 8.07 (ddd, *J* = 8.2, 1.2, 0.6 Hz, 1H), 7.81 (ddd, *J* = 8.0, 1.3, 0.7 Hz, 1H), 7.69 (d, *J* = 7.9 Hz, 1H), 7.47 (ddd, *J* = 8.3, 7.2, 1.3 Hz, 1H), 7.35 (ddd, *J* = 8.3, 7.2, 1.2 Hz, 1H), 7.15 (d, *J* = 7.9 Hz, 1H), 6.98 (s, 1H), 4.94 (ddd, *J* = 8.4, 7.3, 5.7 Hz, 1H), 4.66 (dt, *J* = 9.0, 5.9 Hz, 1H), 3.87 (ddd, *J* = 11.0, 8.4, 6.0 Hz, 1H), 3.23 (ddd, *J* = 11.0, 9.0, 7.3 Hz, 1H), 2.35 (s, 3H), 2.11 (s, 3H). ^13^C NMR (101 MHz, CDCl_3_) δ 175.91 (C), 153.52 (C), 139.02 (C), 138.23 (C), 134.80 (C), 132.18 (CH), 126.54 (CH), 126.03 (CH), 125.27 (CH), 124.93 (CH), 123.71 (CH), 121.88 (CH), 121.23(C), 87.80 (C), 66.71 (CH_2_), 33.60 (CH_2_), 21.20 (CH_3_), 19.85 (CH_3_). HPLC: *t*_R_ 8.921 min, >95% purity (214 and 254 nm). HRMS (*m*/*z*): C_18_H_17_NOS requires [M+H]^+^ 296.1104; found 296.1105.

*1-(Benzo[d]thiazol-2-yl)-1-(2,4-dimethylphenyl)prop-2-en-1-ol (* **7b** *)*

^1^H NMR (401 MHz, CDCl_3_) δ 8.02 (ddd, *J* = 8.2, 1.2, 0.6 Hz, 1H), 7.83 (ddd, *J* = 7.9, 1.3, 0.6 Hz, 1H), 7.48 (ddd, *J* = 8.3, 7.2, 1.3 Hz, 1H), 7.43–7.34 (m, 2H), 7.01 (d, *J* = 8.5 Hz, 2H), 6.64 (dd, *J* = 17.1, 10.5 Hz, 1H), 5.47 (dd, *J* = 17.1, 0.9 Hz, 1H), 5.41 (dd, *J* = 10.5, 0.9 Hz, 1H), 3.81 (s, 1H), 2.32 (s, 3H), 2.16 (s, 3H). ^13^C NMR (101 MHz, CDCl_3_) δ 177.71 (C), 152.44 (C), 141.66 (CH), 138.72 (C), 137.97 (C), 137.92 (C), 136.01 (C), 133.54 (CH), 128.04 (CH), 126.29 (2C), 125.39 (CH), 123.34 (CH), 121.89 (CH), 115.57 (CH_2_), 79.89 (C), 21.32 (CH_3_), 21.08 (CH_3_). HRMS (*m*/*z*): C_18_H_17_NOS requires [M+H]^+^ 296.1104; found 296.1111.

*Synthesis of 2-(1-(2,4-Dimethylphenyl)-1-methoxyethyl)benzo[d]thiazole (* **8** *)*

Synthesis was modified from the literature procedure [19]. A solution of **2** (80.2 mg, 0.28 mmol) in THF (5 mL) was added dropwise to a suspension of NaH (from 55% dispersion in oil, 2 equiv.) in THF. When hydrogen evolution had ceased, methyl iodide (2.5 equiv.) was added to the solution, and it was stirred for 5 h. The crude mixture was quenched with NH_4_Cl (15 mL), extracted with ether (3 × 10 mL), washed with brine, dried over Na_2_SO_4_ and evaporated under vacuum. The resulting crude was purified via preparative TLC (20:1 toluene:acetone) to afford the desired product as a white solid (40%). ^1^H NMR (401 MHz, CDCl_3_) *δ* 7.97 (ddd, *J* = 8.2, 1.3, 0.7 Hz, 1H), 7.86 (ddd, *J* = 7.9, 1.3, 0.7 Hz, 1H), 7.50 (d, *J* = 8.0 Hz, 1H), 7.42 (ddd, *J* = 8.2, 7.2, 1.3 Hz, 1H), 7.34 (ddd, *J* = 8.3, 7.2, 1.2 Hz, 1H), 7.07 (d, *J* = 8.0 Hz, 1H), 6.96 (s, 1H), 3.21 (s, 3H), 2.33 (s, 3H), 2.08 (s, 3H), 2.04 (s, 3H). ^13^C NMR (101 MHz, CDCl_3_) *δ* 179.01 (C), 153.08 (C), 138.28 (C), 137.73 (C), 136.60 (C), 135.55 (C), 133.26 (CH), 128.00 (CH), 126.44 (CH), 125.77 (CH), 124.94 (CH), 123.46 (CH), 121.69 (CH), 82.31 (C), 77.16 (CH_3_), 50.53 (CH_3_), 25.32 (CH_3_), 21.10 (CH_3_). HPLC: *t*_R_ 6.388 min, >95% purity (214 and 254 nm). HRMS (*m*/*z*): C_18_H_19_NOS requires [M+H]^+^ 298.1260; found 298.1273.

*Synthesis of 2-(1-(2,4-Dimethylphenyl)-1-fluoroethyl)benzo[d]thiazole (* **9** *)*

Synthesis was modified from the literature procedure [20]. A solution of **2** (53 mg, 0.187 mmol) in anhydrous DCM was cooled to −78 °C, followed by the addition of 1,8-diazabicyclo[5.4.0]undec-7-ene (DBU) (1.5 equiv.) and XtalFluor-M (1.5 equiv.) under nitrogen. The reaction mixture was allowed to warm to room temperature and stirred for 24 h. The resulting mixture was quenched with a 5% aqueous sodium bicarbonate (5 mL) solution and extracted with DCM (3 × 5 mL), dried over Na_2_SO_4_, filtered and concentrated under reduced pressure. The resulting crude was purified via silica gel column chromatography (straight toluene, then 20:1 toluene:EtOAc) to afford the product as a clear paste (11.5%). ^1^H NMR (401 MHz, CDCl_3_) *δ* 8.05 (ddd, *J* = 8.2, 1.2, 0.7 Hz, 1H), 7.87 (ddd, *J* = 8.0, 1.3, 0.6 Hz, 1H), 7.51–7.46 (m, 1H), 7.46–7.43 (m, 1H), 7.40 (ddd, *J* = 7.9, 7.2, 1.2 Hz, 1H), 7.06 (d, *J* = 8.0 Hz, 1H), 7.00 (s, 1H), 2.33 (s, 3H), 2.27 (d, *J* = 23.0 Hz, 3H), 2.21 (d, *J* = 2.6 Hz, 3H). ^19^F NMR (376 MHz, CDCl_3_) *δ* -96.78. ^13^C NMR (101 MHz, CDCl_3_) *δ* 174.21 (d, *J* = 29.6 Hz, C), 152.91 (C), 138.94 (d, *J* = 1.4 Hz, C), 136.55 (d, J = 21.1 Hz, C), 136.29 (d, *J* = 1.9 Hz, C), 135.82 (C), 133.26 (CH), 126.60 (CH), 126.21 (CH), 126.08 (d, *J* = 9.7 Hz, CH), 125.60 (CH), 123.88 (CH), 121.85 (CH), 97.80 (d, *J* = 172.2 Hz, C), 27.39 (d, *J* = 25.5 Hz, CH_3_), 21.12 (d, *J* = 5.2 Hz, CH_3_), 21.08 (CH_3_). HPLC: *t*_R_ 6.400 min, >95% purity (214 and 254 nm). HRMS (*m*/*z*): C_17_H_16_FNS requires [M+H]^+^ 286.1060; found 286.1073.

### 3.3. Pharmacological Evaluation of Compounds

#### 3.3.1. Functional Assay—Ca^2+^_i_ Mobilisation

Ca^2+^_i_ mobilisation assays were performed as previously described [28,29]. Specifically, FlpIn-TRex-HEK293-CaSR stable cell lines were seeded at 80,000 cells/well in poly-D-lysine (50 μg/mL)-coated clear 96-well plates and incubated overnight at 37 °C, 5% CO_2_, in the presence of 100 ng/mL tetracycline to induce CaSR expression. Cells were washed in assay buffer (150 mM NaCl, 2.6 mM KCl, 1.18 mM MgCl_2_, 10 mM D-glucose, 10 mM HEPES, 0.1 mM CaCl_2_, 0.5% BSA, 4 mM probenecid at pH 7.4) and loaded with 1 μM Fluo-8^®^ AM in assay buffer for 1 h. The agonist and PAMs (0, 0.03, 0.1, 0.3, 1, 3, 10 or 30 μM) were co-added, and measurements of Ca^2+^_i_ mobilisation were performed in duplicate at 37 °C using a Flexstation 1 or 3 (Molecular Devices, Sunnyvale, CA, USA). Fluorescence was detected for 20 s to establish a baseline response and then a further 60 s at 490 nm excitation and 520 nm emission. Data were normalised to the percentage of ionomycin response. Ionomycin (1 μM) was used as an internal assay control.

#### 3.3.2. Data Statistical Analysis

Data describing the interaction between Ca^2+^_o_ and the PAMs were fitted to the following variant of the operational model of cooperative agonism and allosteric modulation, which was modified to accommodate the presence of the ambient agonist [27,35]:

(1) $$\mathrm{Effect}\;=\;\frac{{E_{m}\left({\left[A+C\right]}^{nB}\left(K_{B}+\alpha \beta \left[B\right]\right)+\tau_{B}\left[B\right]{\left[{\mathrm{EC}}_{50}\right]}^{nB}\right)}^{nT}}{{{{[\mathrm{EC}}_{50}]}^{nBnT}{\left(K_{B}+[B]\right)}^{nT}+\left({\left[A+C\right]}^{nB}\left(K_{B}+\alpha \beta \;[B]\right)+\tau_{B\;}\left[B\right]{\left[{\mathrm{EC}}_{50}\right]}^{nB}\right)}^{nT}}$$

where EC_50_ is the agonist concentration (M) that produces a half maximal response; *K*_B_ is the affinity of the allosteric ligand; *τ*_B_ is the efficacy of the allosteric ligand; *α* and *β* describe the allosteric effects on orthosteric agonist affinity and efficacy, respectively; [A] and [B] are the orthosteric agonist and allosteric ligand molar concentrations, respectively; *E*_m_ is the maximal system response; *nT* is the slope of the transducer function linking agonist receptor occupancy to response and constrained to unity; *nB* is the slope of agonist binding linking agonist concentration to occupancy; and [C] is the ambient Ca^2+^_o_ concentration present in the assay buffer.

All non-linear regression analysis was performed in GraphPad Prism 10.4.1 (GraphPad Software, San Diego, CA, USA). Affinity, cooperativity and efficacy parameters were estimated as logarithms [36]. A one-way ANOVA with Dunnett’s multiple-comparison post hoc test was used to determine statistical differences between PAM p*K*_B_, Log*αβ* and Log*τ*_B_, where significance was defined as *p* < 0.05.

### 3.4. Computational Modelling of Compounds

Structural information obtained from the Cryo-EM PDBID:7M3G [30] was used as the basis for docking. Docking was performed using ICM, where the candidate was placed in the 7TM binding pocket that contained evocalcet. PAMs were initially placed in the centre of the presumed binding site before being extensively sampled by biased probability Monte Carlo sampling in internal coordinates. Therefore, the hydrogen bonding, van der Waals, and hydrophobic and electrostatic potential of the CaSR 7TM binding cavity were calculated to create a ‘grid potential map’, which was used to score the binding potential of randomised conformations of the PAM. Binding scores were calculated as described previously [37]. Ten poses with the lowest calculated binding scores were retained for each PAM. These poses were further refined using a ‘flexible receptor’ approach, by undertaking biased probability Monte Carlo optimisation of receptor residue sidechains [38], while simultaneously sampling PAM shape and position by Monte Carlo randomisation. Final results were assessed using a combination of ICM docking scores and calculated binding energies [39,40], coupled with manual inspection for agreement with mutagenesis data for AC265347 (**1**) and published SAR data [5,29].

## 4. Conclusions

A series of novel analogues generated from AC265347 (**1**) were synthesised as racemates in this SAR study. The library of analogues provided insight into the importance of the tertiary alcohol feature and concomitant H-bonding profile at the stereogenic centre to the *ago*-PAM profile of **1**. Structural modifications to the hydroxy motif afforded compounds exhibiting a ‘pure’ PAM profile (epoxide **6**, oxetane **7** and fluorinated compound **9**) with a range of affinities and cooperativities and devoid of intrinsic agonism. The methoxy analogue **8** could not reliably be fitted with the operational model of cooperative agonism and allosterism, and therefore affinity, cooperativity and intrinsic agonism could not be estimated. This abolition of agonism demonstrated a close correlation between intrinsic agonism and the hydroxy group, perhaps suggesting the role of the hydroxy group in allosteric CaSR agonism. Moreover, alterations in spatial orientation (epoxide **6** and oxetane **7**) and replacement of hydroxy (fluoro analogue **9**) that can interrupt the potential non-covalent intramolecular interaction between the benzothiazole sulphur and alcohol oxygen atoms led to a diminution of agonist activity at the CaSR. The study extends the previous work by Gustaffson et al. exploring the significance of the hydroxy group for CaSR allosteric activity. The authors recognise and acknowledge a limitation of this study on the basis that the compounds were tested as racemates rather than individual enantiomers, and therefore, care should be exercised in the absolute interpretation of biochemical data. This is, however, consistent with the reference compound AC265347 (**1**), which is commercially available as a racemate and extensively represented as such in the literature. Furthermore, the SAR investigation herein has enriched the key structural features for activity at the CaSR, and identified novel chemical tools to ascertain the therapeutic potential of AC265367-like PAMs devoid of intrinsic agonism, thereby providing a new promise and purpose for a previously abandoned drug candidate.

## Data Availability

The datasets presented in this article are not readily available because the data are part of an ongoing study. Requests to access the datasets should be directed to the corresponding authors via email.

## Supplementary Materials

The following supporting information can be downloaded at: https://www.mdpi.com/article/10.3390/ijms26062580/s1. References [4,27] are cited in the supplementary materials.
