# Supplementary material for: Next-Generation Analogues of AC265347 as Positive Allosteric Modulators of the Calcium-Sensing Receptor: Pharmacological Investigation of Structural Modifications at the Stereogenic Centre
Source: Int J Mol Sci. 2025 Mar 13;26(6):2580. doi: 10.3390/ijms26062580 (PMC11942566; doi:10.3390/ijms26062580)
Supplement: Supplementary file 1 [file ijms-26-02580-s001.zip › ijms-3408822-supplementary.pdf]

SUPPORTING INFORMATION

Next-Generation Analogues of AC265347 as Positive Allosteric Modulators of the Calcium-Sensing Receptor: Pharmacological Investigation of Structural Modifications at the Stereogenic Centre

Le Vi Dinh [1,2], Jesse Dangerfield [1,2], Aaron DeBono [1,2], Andrew N. Keller [2], Tracy M. Josephs [2,3], Karen J. Gregory [2,3,*], Katie Leach [2,3,*] and Ben Capuano [1,*]

Table of Contents

Characterization and purity data for epoxide compound **6**

^{1}H NMR and ^{13}C NMR spectra, HPLC trace and HRMS data S2

Characterization and purity data for oxetane compound **7**

^{1}H NMR and ^{13}C NMR spectra, HPLC trace and HRMS data S4

Characterization and purity data for allylic alcohol compound **7b**

^{1}H NMR and ^{13}C NMR spectra, HPLC trace and HRMS data S6

Characterization and purity data for methoxy compound **8**

^{1}H NMR and ^{13}C NMR spectra, HPLC trace and HRMS data S8

Characterization and purity data for fluoro compound **9**

^{1}H NMR and ^{13}C NMR spectra, HPLC trace and HRMS data S10

Pharmacology Data S12

References S12

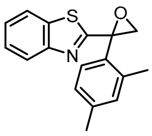

Compound **6**

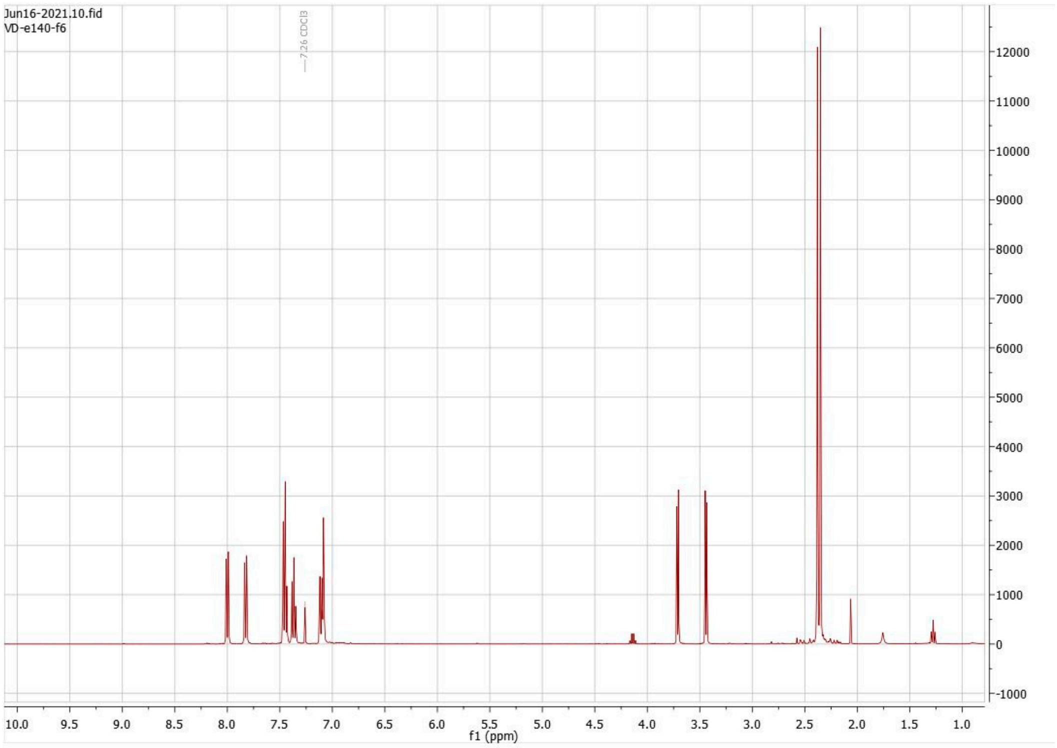

^{1}H NMR spectrum of **6**

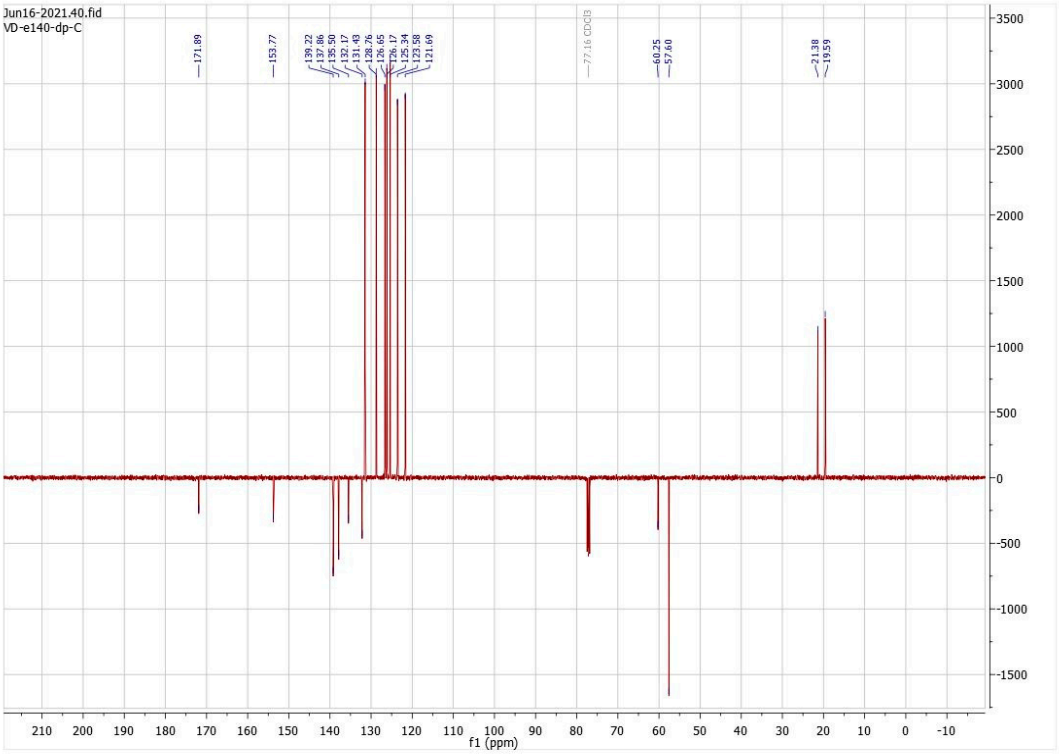

^{13}C NMR spectrum of **6**

```
PMP1, Solvent A         : Water 0.1% TFA
PMP1, Solvent B         : ACN 0.1% TFA
```

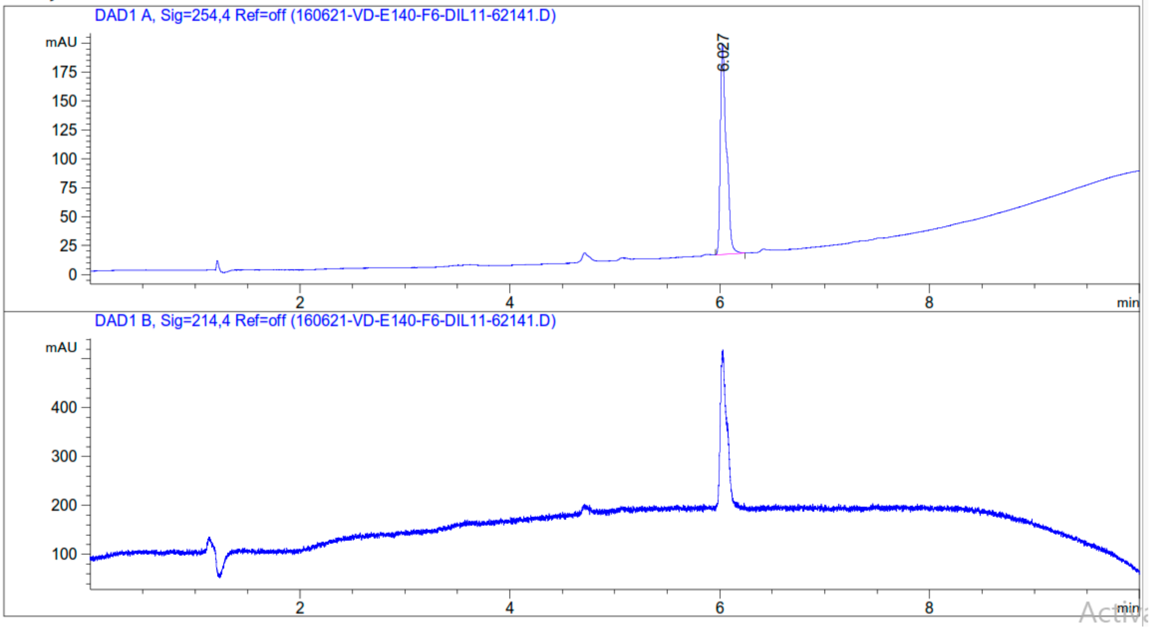

HPLC trace of **6** (upper, 254 nm; lower 214 nm)

MS Spectrum Peak List

m/z	Calc *m/z*	Diff(ppm)	z	Abund	Formula	Ion
282.0959	282.0947	-4.08	1	5305034.37	C17H15NOS	(M+H)+
304.0767	304.0767	-0.02	1	11103.91	C17H15NOS	(M+Na)+
585.1642	585.1641	-0.15	1	29356.7	C17H15NOS	(2M+Na)+

HRMS data for **6**

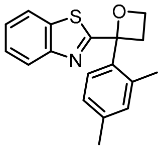

Compound **7**

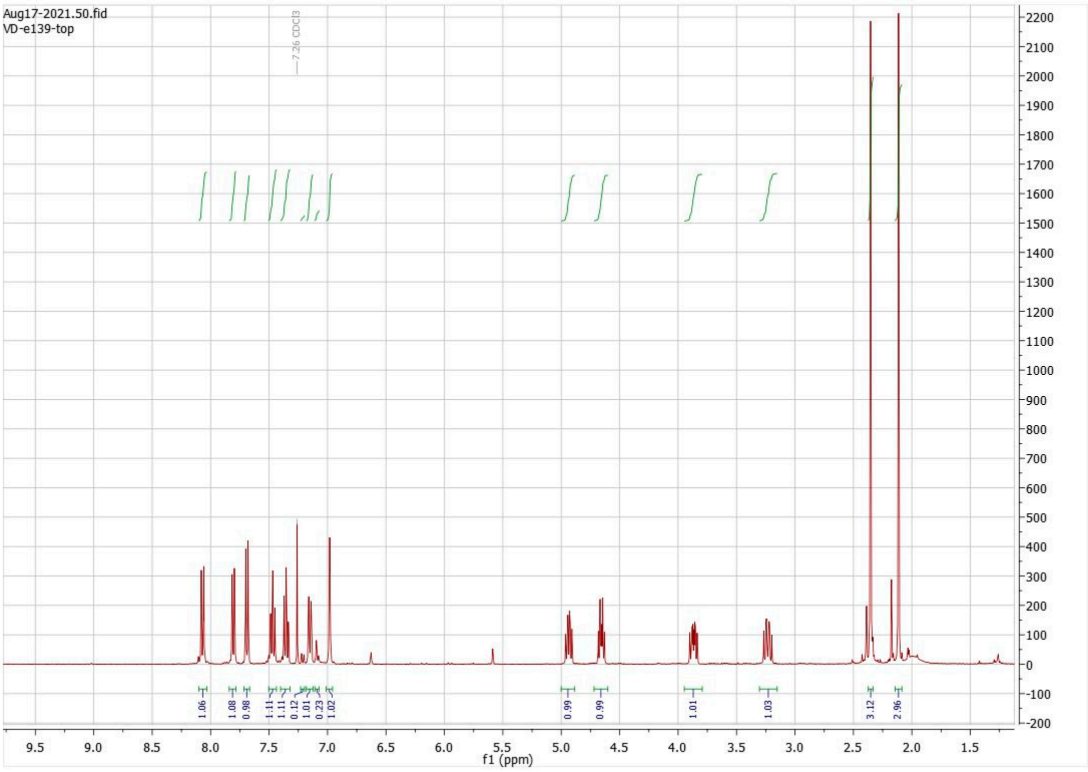

^{1}H NMR spectrum of **7**

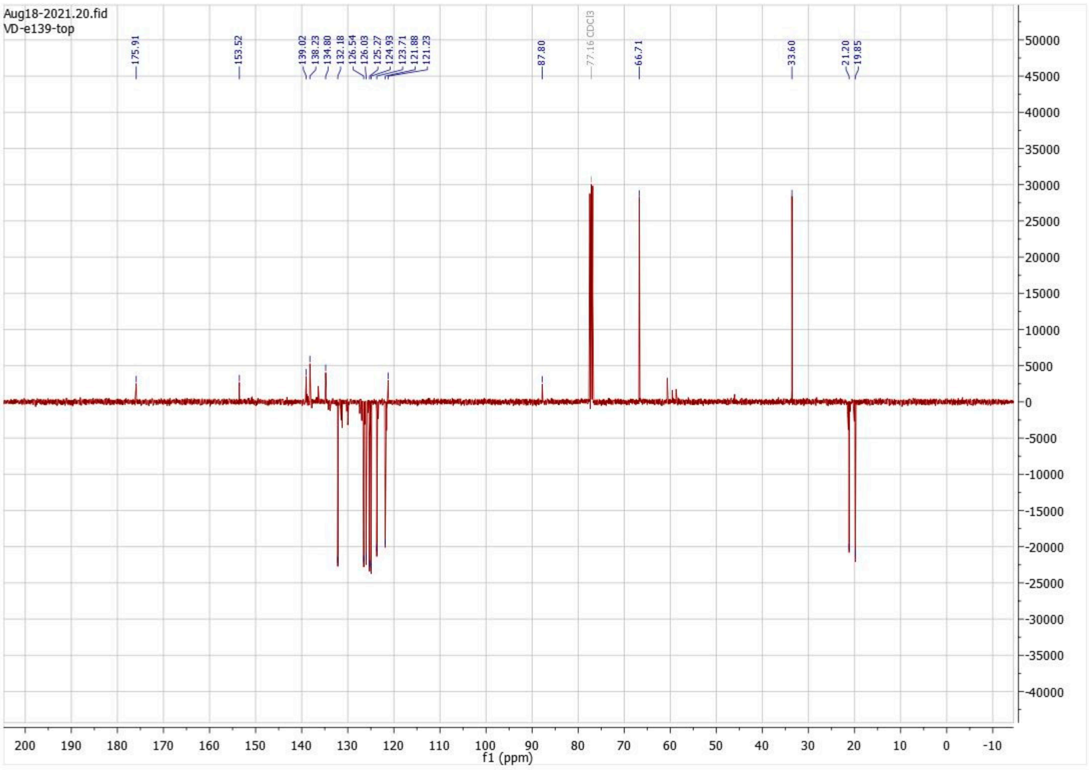

^{13}C NMR spectrum of **7**

```
Solvent Description      :
PMP1, Solvent A          : Water 0.1% TFA
PMP1, Solvent B          : ACN 0.1% TFA
```

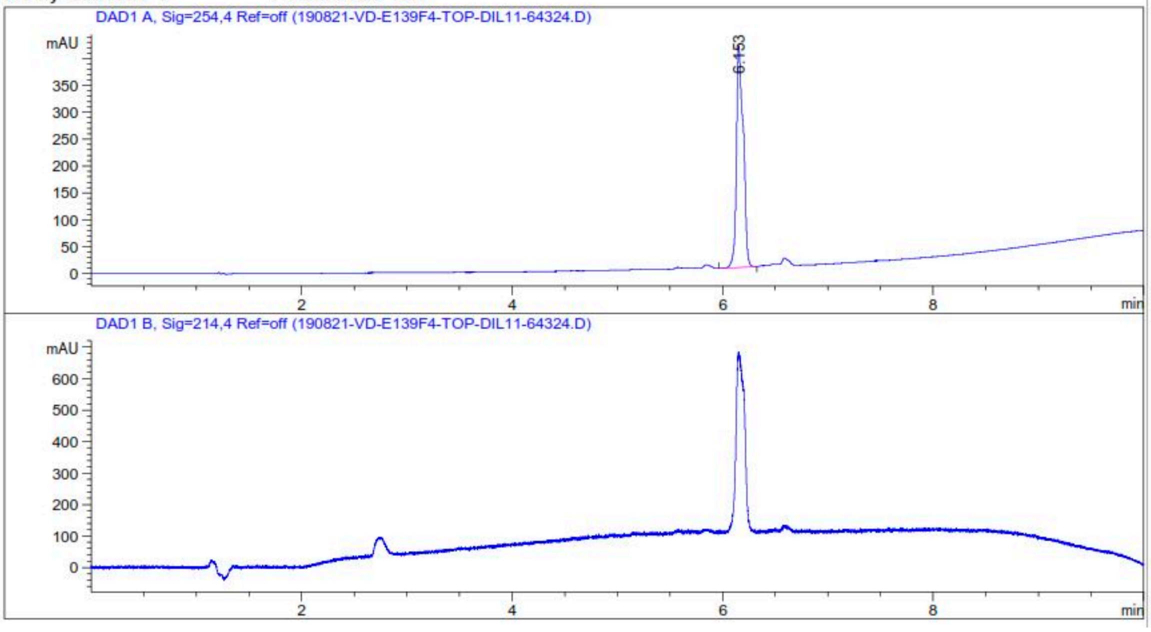

HPLC trace of **7** (upper, 254 nm; lower 214 nm)

Spectrum Peaks (Max. 3)

m/z	m/z (Calc)	Diff (ppm)	Abund	Height %	Height % (Calc)	Ion Species	Z
296.1105	296.1104	0.63	761988	100.00	100.00	(M+H)+	1
297.1135	297.1135	0.19	153120	20.09	20.87	(M+H)+	1
318.0928	318.0923	1.45	7653	100.00	100.00	(M+Na)+	1

HRMS data for **7**

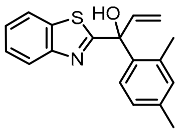

Compound **7b** – by-product

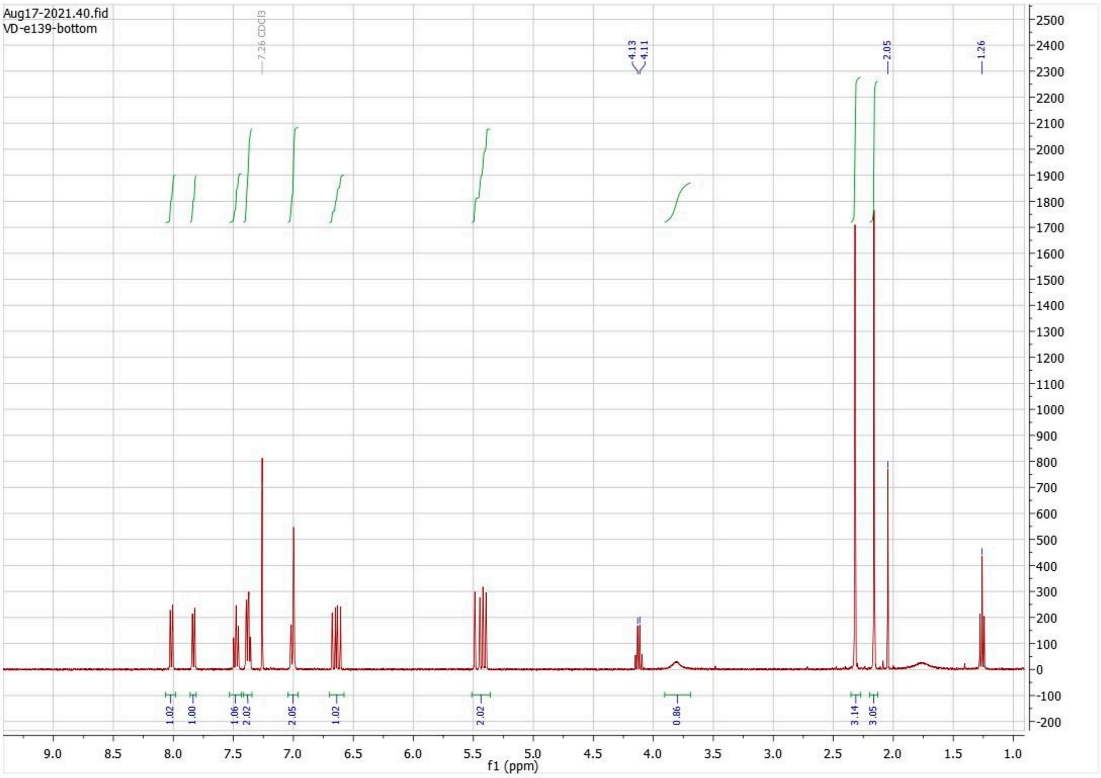

^{1}H NMR spectrum of **7b**

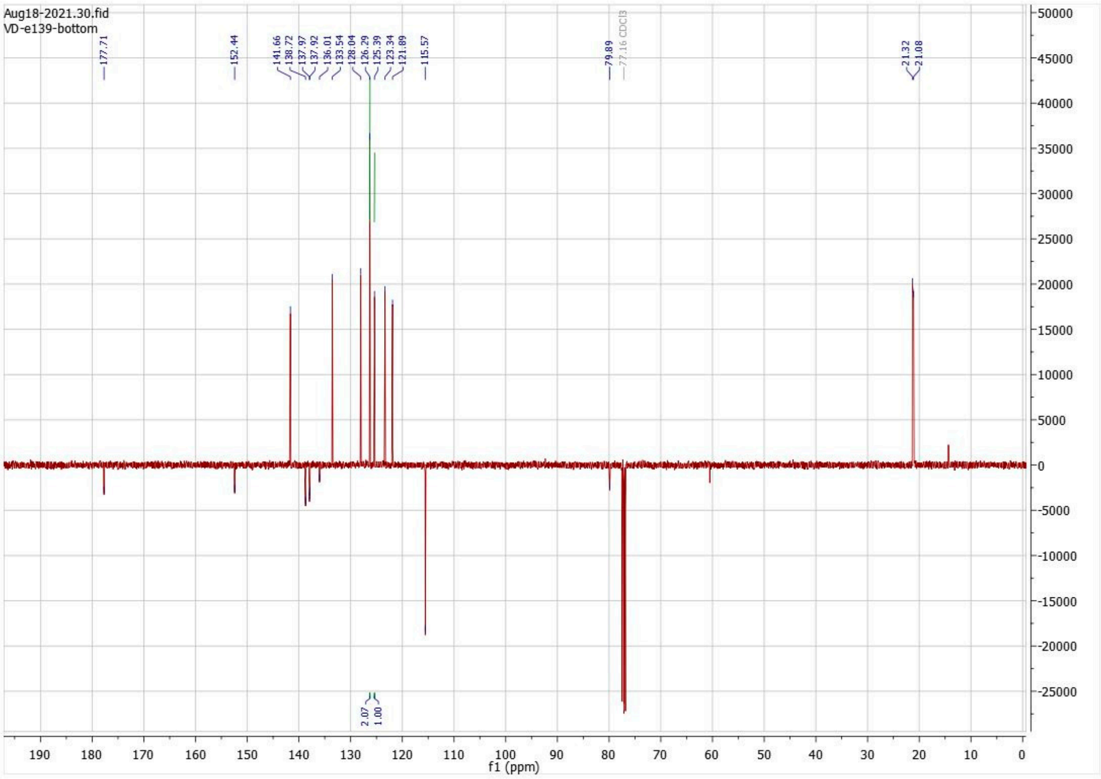

^{13}C NMR spectrum of **7b**

```
Solvent Description      :
PMP1, Solvent A          : Water 0.1% TFA
PMP1, Solvent A          :
PMP1, Solvent B          : ACN 0.1% TFA
PMP1, Solvent B          :
```

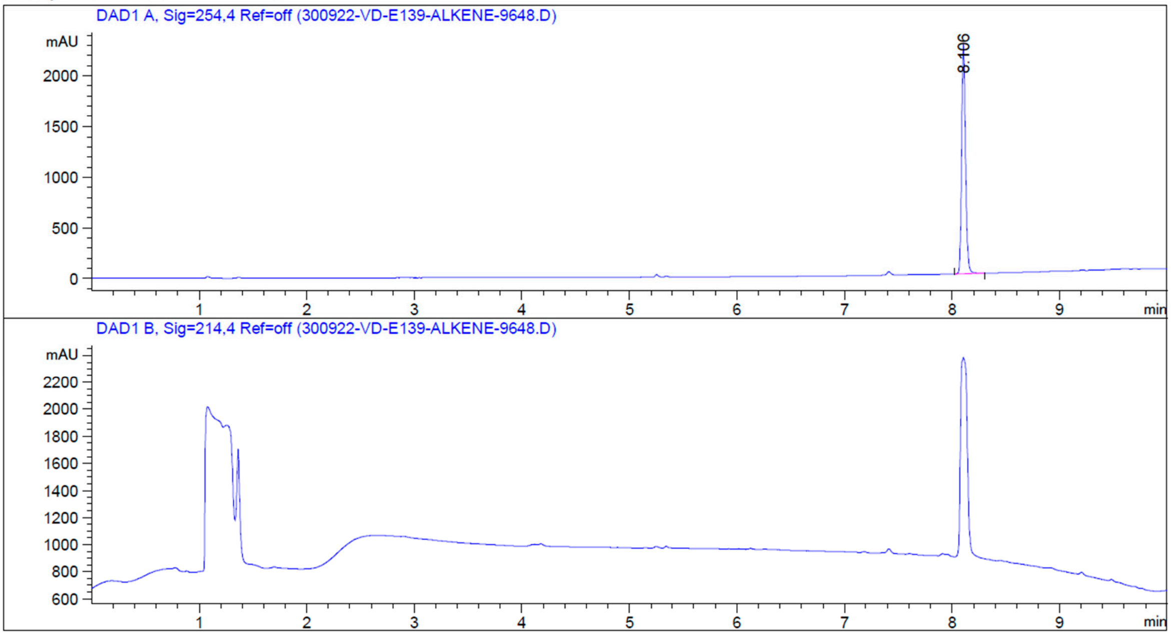

HPLC trace of **7b** (upper, 254 nm; lower 214 nm)

Spectrum Peaks (Max. 3)

m/z	m/z (Calc)	Diff (ppm)	Abund	Height %	Height % (Calc)	Ion Species	Z
296.1111	296.1104	2.44	307342	100.00	100.00	(M+H)+	1
297.1136	297.1135	0.51	61015	19.85	20.87	(M+H)+	1
298.1099	298.1096	1.06	18011	5.86	6.75	(M+H)+	1

HRMS data for **7b**

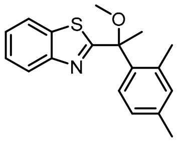

Compound **8**

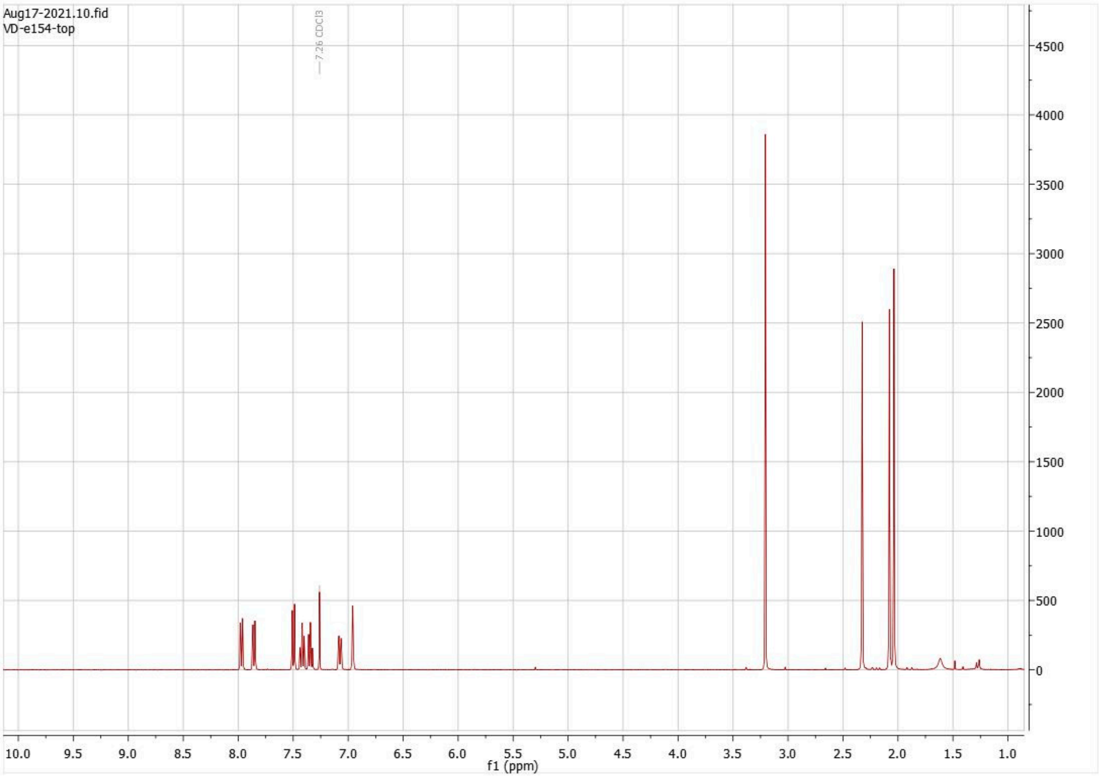

^{1}H NMR spectrum of **8**

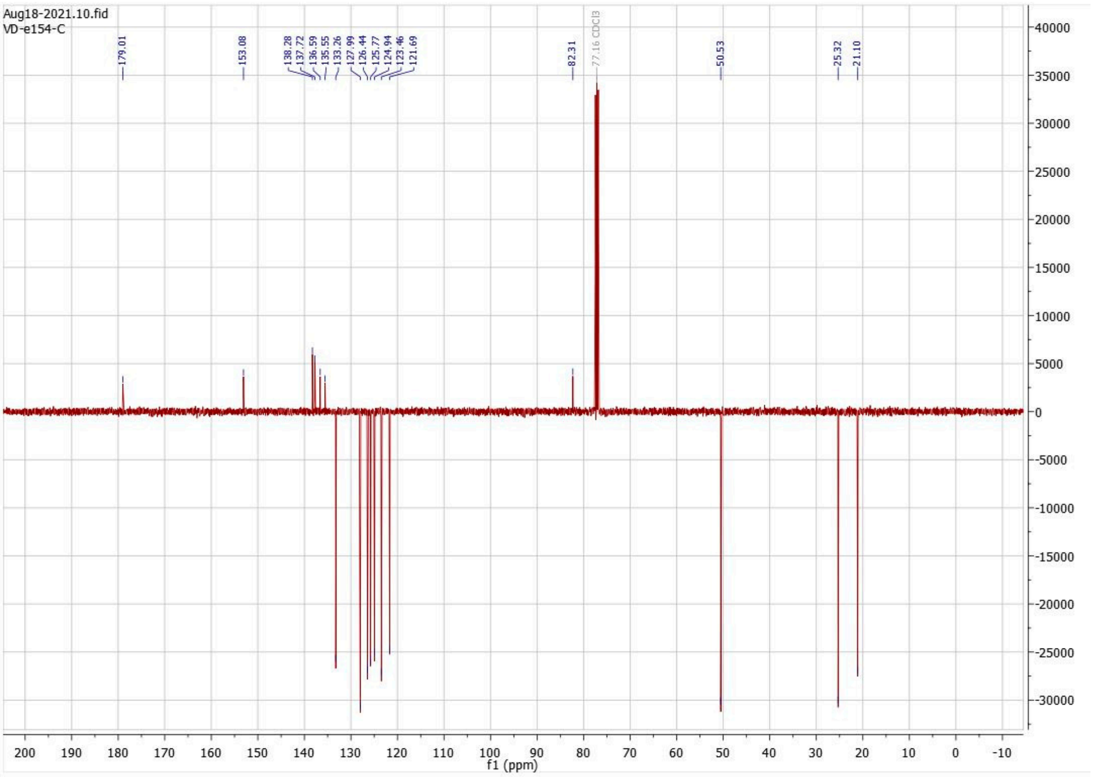

^{13}C NMR spectrum of **8**

```
Solvent Description     :
PMP1, Solvent A         : Water 0.1% TFA
PMP1, Solvent B         : ACN 0.1% TFA
```

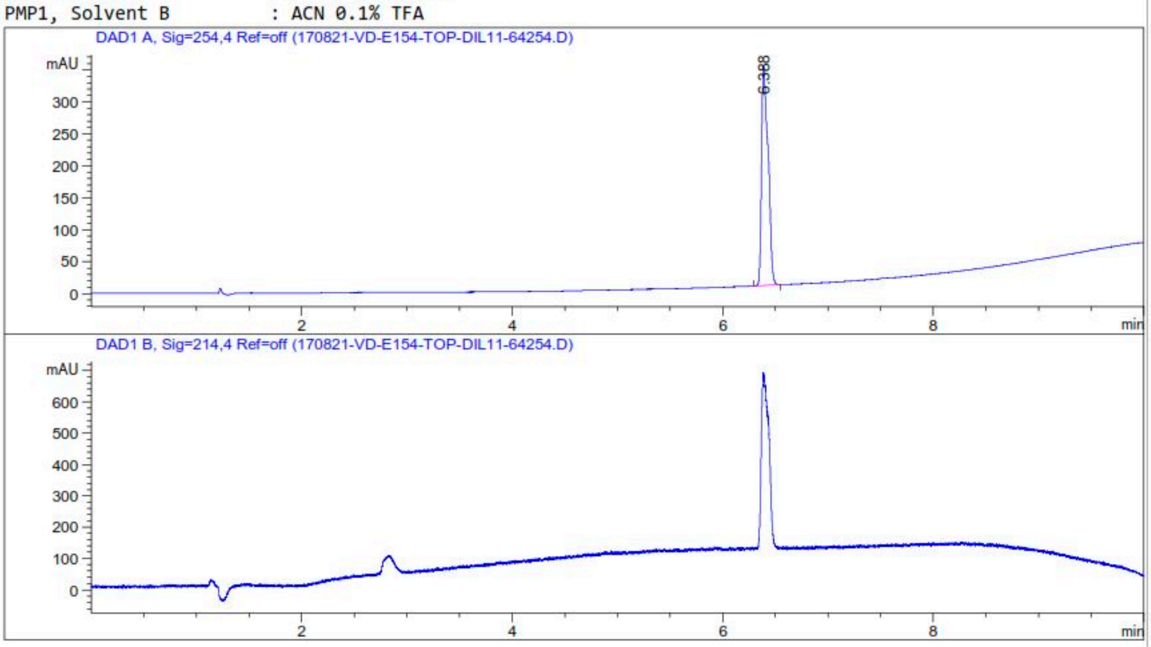

HPLC trace of **8** (upper, 254 nm; lower 214 nm)

MS Spectrum Peak List

m/z	Calc *m/z*	Diff(ppm)	z	Abund	Formula	Ion
298.1273	298.126	-4.2	1	7777539.15	C18H19NOS	(M+H)+
320.1078	320.108	0.39	1	33207.42	C18H19NOS	(M+Na)+
617.2269	617.2267	-0.33	1	31256.68	C18H19NOS	(2M+Na)+

HRMS data for **8**

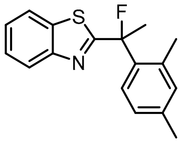

Compound **9**

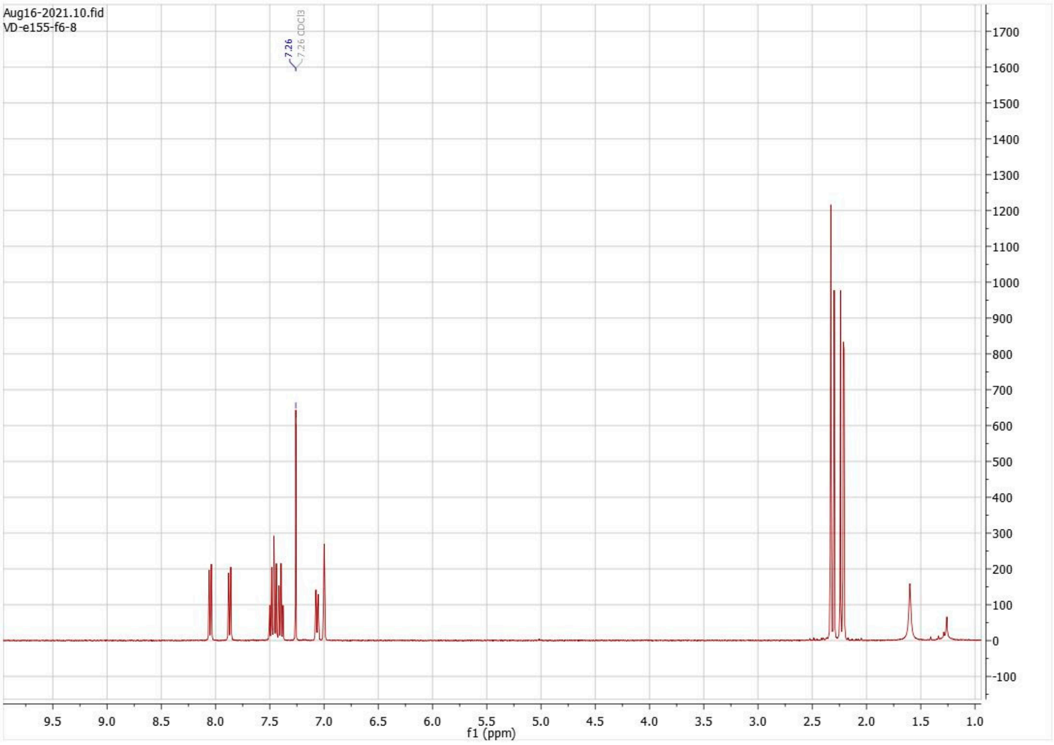

^{1}H NMR spectrum of **9**

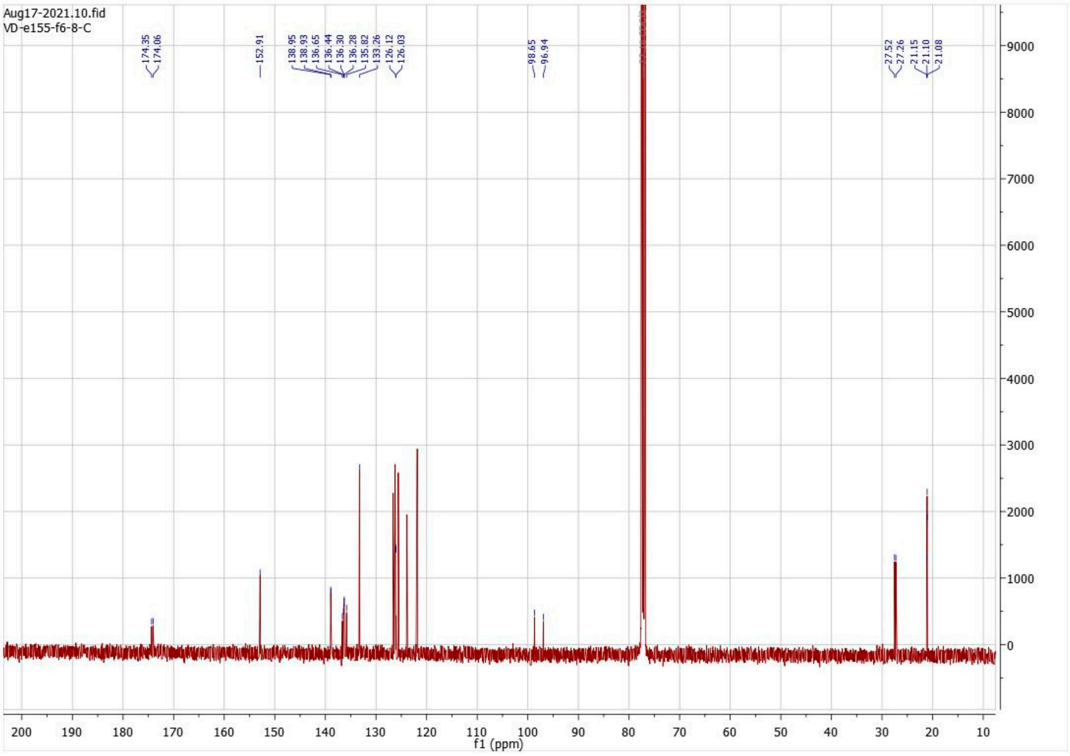

^{13}C NMR spectrum of **9**

```
Solvent Description      :
PMP1, Solvent A          : Water 0.1% TFA
PMP1, Solvent B          : ACN 0.1% TFA
```

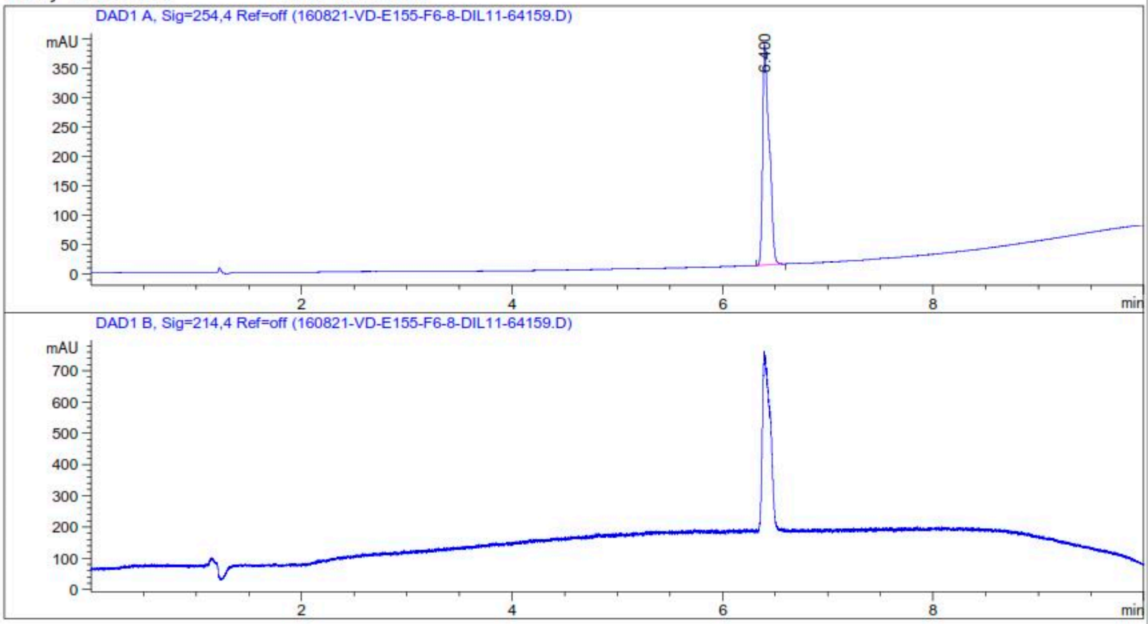

HPLC trace of **9** (upper, 254 nm; lower 214 nm)

MS Spectrum Peak List

m/z	Calc *m/z*	Diff(ppm)	z	Abund	Formula	Ion
286.1073	286.106	-4.38	1	1782783.21	C17H16FNS	(M+H)+
287.1104	287.1091	-4.4	1	335051.78	C17H16FNS	(M+H)+
308.0865	308.088	4.88	1	3605.38	C17H16FNS	(M+Na)+

HRMS data for **9**

Pharmacology Data

Supplementary Table 1: Summary of parameters from fitting operational model of comparative cooperative agonism and allosterism to collated data from multiple independent experiments (*N*). Data represent the mean ± SEM.

**Entry**	Ca^{2+} pEC_{50} [a]	n^{B} #	basal	E_m [b]	*N*
AC265347 (**1**)[c]	3.16 ± 0.02	2.6 ± 0.2	-2.6 ± 2.3	96.8 ± 1.5	5
**5**[c]	3.53 ± 0.04	2.7 ± 0.5	-14.8 ± 9.9	103.8 ± 2.3	5
**6**	3.39 ± 0.03	3.5 ± 0.5	-7.7 ± 4.5	124.7 ± 2.9	3-7
**7**	3.40 ± 0.01	4.3 ± 0.3	-2.8 ± 1.4	86.4 ± 1.0	3-7
**7b**	3.42 ± 0.03	4.8 ± 0.8	-4.5 ± 3.1	83.8 ± 1.4	4
**9**	3.39 ± 0.02	5.6 ± 0.7	-2.4 ± 2.1	87.1 ± 1.5	4

[a]A simplified version of the operational model of allosterism with cooperative agonism equation was used, where pEC_{50} is the negative logarithm of the Ca^{2+} concentration required to elicit a half-maximal response. In deriving these estimates, the buffer concentration of Ca^{2+} (0.1mM) was factored in.

[#]binding slope factor to account for cooperative Ca^{2+} binding to the CaSR

[b]maximal system response expressed as % of the maximal peak response to ionomycin

[c]reanalysis of previously published data sets [1,2]. For these analyses all parameters were allowed to float and derived from global fit of collated experiments as summarised herein.

References

[1] Dinh, L.V.; DeBono, A.; Keller, A.N.; Josephs, T.M.; Gregory, K.J.; Leach, K.; Capuano, B. Development of AC265347-Inspired Calcium-Sensing Receptor Ago-Positive Allosteric Modulators. *ChemMedChem* **2021**, *16*, 3451-3462.

[2] Gregory, K.J.; Giraldo, J.; Diao, J.; Christopoulos, A.; Leach, K. Evaluation of Operational Models of Agonism and Allosterism at Receptors with Multiple Orthosteric Binding Sites. *Mol Pharmacol* **2020**, *97*, 35-45.
